# Transcriptional Homeostasis of a Mangrove Species, *Ceriops tagal*, in Saline Environments, as Revealed by Microarray Analysis

**DOI:** 10.1371/journal.pone.0036499

**Published:** 2012-05-04

**Authors:** Shan Liang, Lu Fang, Renchao Zhou, Tian Tang, Shulin Deng, Suisui Dong, Yelin Huang, Cairong Zhong, Suhua Shi

**Affiliations:** 1 State Key Laboratory of Biocontrol and Guangdong Key Laboratory of Plant Resources, Sun Yat-Sen University, Guangzhou, China; 2 Guangdong Key Lab of Biotechnology for Plant Development, South China Normal University, Guangzhou, China; 3 Hainan Dongzhai Harbor National Nature Reserve, Hainan, China; Glasgow Caledonian University, United Kingdom

## Abstract

**Background:**

Differential responses to the environmental stresses at the level of transcription play a critical role in adaptation. Mangrove species compose a dominant community in intertidal zones and form dense forests at the sea-land interface, and although the anatomical and physiological features associated with their salt-tolerant lifestyles have been well characterized, little is known about the impact of transcriptional phenotypes on their adaptation to these saline environments.

**Methodology and Principal findings:**

We report the time-course transcript profiles in the roots of a true mangrove species, *Ceriops tagal*, as revealed by a series of microarray experiments. The expression of a total of 432 transcripts changed significantly in the roots of *C. tagal* under salt shock, of which 83 had a more than 2-fold change and were further assembled into 59 unigenes. Global transcription was stable at the early stage of salt stress and then was gradually dysregulated with the increased duration of the stress. Importantly, a pair-wise comparison of predicted homologous gene pairs revealed that the transcriptional regulations of most of the differentially expressed genes were highly divergent in *C. tagal* from that in salt-sensitive species, *Arabidopsis thaliana*.

**Conclusions/Significance:**

This work suggests that transcriptional homeostasis and specific transcriptional regulation are major events in the roots of *C. tagal* when subjected to salt shock, which could contribute to the establishment of adaptation to saline environments and, thus, facilitate the salt-tolerant lifestyle of this mangrove species. Furthermore, the candidate genes underlying the adaptation were identified through comparative analyses. This study provides a foundation for dissecting the genetic basis of the adaptation of mangroves to intertidal environments.

## Introduction

Gene expression is modulated by environmental factors and is, thus, a good molecular phenotypic marker for illustrating the interaction between genotypes and environmental factors and can also be used for identifying the so-called “plasticity genes” that determine phenotypic-plasticity-derived evolutionary adaptations [1]–[3]. Transcriptional variations across species or individuals often lead to extraordinary evolutionary consequences, such as the establishment of adaptation or speciation [4], whereas selection plays a major role in the dynamics of transcript abundance alterations such as that revealed by Denver et al [5]. Due to technological limitations and, more importantly, the lack of suitable data for ecological and evolutionary analyses, our understanding of the connections between changes in expression and evolution are confined to a few regulatory pathways in a handful of model organisms [4]. However, the advent of the microarray technique has provided ample opportunities to monitor changes in gene expression and to conduct in-depth ecological and evolutionary analyses in non-model organisms.

Serving as selection pressures, exogenous environmental stresses may lead to ecological divergence and play important roles in adaptation [6], an effect that is commonly observed in humans, animals and certain plants at both levels of gene sequence and gene expression [7]–[14]. However, in-depth studies of the evolutionary significance of environmental stresses in plants, especially non-model species, are fairly rare to date, mainly due to the difficulties in integrating the analysis of stress-induced responses and the assessment of evolutionary changes. In a previous study, Bressan [15] hypothesized that evolutionary divergence and adaptation to an extreme lifestyle in plants may have led to the appearance of novel gene combinations that support tolerance. Thus, it is intriguing to identify such genes or gene sets that are associated with the stress-related divergence between species that differ strikingly in stress tolerance. Although salt tolerance in model plants has attracted the attention of researchers for years and the knowledge of salt-induced responses has been enriched by exploring physiological and molecular mechanisms [16]–[23], studies emphasizing the loci underlying salt adaptation from an evolutionary perspective are rarely reported. Several comparative studies on the transcriptomes of salt-sensitive and salt-tolerant plants offered some novel insights into this issue [21], [24]–[28], providing a linkage between gene expression differences and salt-tolerance capacities.

Mangroves are woody plants that grow along tropical and subtropical coasts and form clumpy stands in intertidal zones [29]–[30]. These trees can tolerate high salinity, though the adaptation competencies vary across species [29], [31]. *Ceriops tagal* (Rhizophoraceae) is a typical true mangrove species that can form rich stands in fields with salinities up to 35‰ [30]. In lab-cultured seedlings, Na^+^ and Cl^−^ may accumulate in the leaves of *C. tagal* when subjected to increasing salinity [32], and also be enriched in the developing propagules [33]. These observations do not fully agree with the ultrafiltration hypothesis [34]–[35] and imply that a combined management and regulation of ion contents may operate in this species.

In this study, we attempted to use the microarray technique to uncover the connection between salt-induced time-course transcript profiling and the salinity-adaptation capability of *C. tagal*. We constructed a customized cDNA microarray containing probes derived from a root cDNA library of *C. tagal* and then monitored the transcript profiles at various time points over a period of salt stress. We identified differentially expressed genes (DEGs) by comparing salt-shocked samples with unstressed controls. Additionally, comparative analyses between *C. tagal* and *Arabidopsis thaliana* were conducted to reveal the transcriptional divergence that may be associated with the salt adaptation of *C. tagal*.

## Materials and Methods

### Plant Growth and Salt Treatment

Propagules of *C. tagal* were collected from Dongzhai Harbor National Nature Reserve, Hainan, China, and planted in culture pots containing a mixture of sand and nutritive soil. The seedlings were grown under a natural photoperiod until the main roots were 1.0–1.5 cm in length. For the cDNA library construction, the seedlings were irrigated with artificial seawater at 15‰ salinity (approximately 250 mM NaCl; salinity = mass of salt/mass of solution). For the microarray analysis, the seedlings were grown in freshwater-irrigated pots until the main roots were 1.0–1.5 cm in length, and were then transferred to another pot and stressed with 500 mM NaCl for 2, 5, 10, 24 and 196 hours (8 days). Unstressed young roots (control sample) were collected in parallel to avoid the possible effects of diurnal or circadian rhythms. A total of 12 seedlings were harvested independently for each time point. The RNA extracted from separate seedlings was mixed into 3 pools as independent biological replicates.

[No specific permits were required for the described field studies in this work.]

### Construction of the cDNA Library

The total RNA was extracted from young roots using the method developed by Fu et al. [36], with minor modifications at the recovery step in which RNase-free filter columns (Sangon) were used instead of the LiCl treatment. All of the RNA extracts were stored at −80°C until use. The quantity and quality of the total RNA were assessed by spectrophotometry and gel electrophoresis after removing the residual DNA by DNase-I digestion. The purified RNA was used as the starting material to construct a directional cDNA library using the Creator™ Smart™ cDNA Library Construction Kit (Clontech), following the manufacturer’s instructions. cDNAs with a size of more than 500 bp were recovered and inserted into the predigested pDNR-LIB vector. The plasmids were transformed into *E. coli* DH5α to generate the primary library.

### Generation of the Customized *C. tagal* cDNA Chip

Independent clones were collected from the root cDNA library, and the cDNA insertion was amplified by hot-start PCR using the following program: hot-start at 94°C and a 4-minute hold for the pre-denaturalization of the template, followed by 32 cycles of 1 minute at 94°C, 30 seconds at 56°C and 1.5 minutes at 72°C. The M13F/M13R primer pair (5′- GTAAAACGACGGCCAGT -3′/5′- AAACAGCTATGACCATGTTCA -3′) was used to prime the reactions. The PCR products were purified using the Multiscreen-PCR 96-well purification system (Millipore), lyophilized and resuspended in 50% DMSO before loading into 384-well plates. A total of 3909 cDNA probes were printed onto a poly-L-lysine-modified slide (Full Moon Biosystems, Inc.) in duplicate using the GeneMachines Omingrid 100 Arrayer (GeneMachine). Each cDNA chip comprised 48 blocks. Within each block, additional cDNA fragments for six *C. tagal* housekeeping genes (coding for actin 11, actin 2, ubiquitin 3, ubiquitin 4, 18 s rRNA and β-tubulin, respectively, to serve as positive controls) and one animal gene (serving as a negative control), the pDNR-LIB vector and 50% DMSO (for background signal monitoring) were also included.

### Microarray Hybridization

The microarray experiments were performed at Shanghai Biochip Co., Ltd, using the customized *C. tagal* cDNA chips. All of the output data are MIAME compliant and have been deposited in the GEO database (GSE30909). In brief, total RNA was extracted from the roots, and linear amplification was performed using the Ambion® MessageAmp™ aRNA Kit (Ambion). The resulting aRNA was then reverse-transcribed in the presence of Cy3-dCTP or Cy5-dCTP (Amersham) to generate labeled cDNA probes. The stressed probes (Cy3-labeled) were paired with the corresponding controls (Cy5-labeled) and co-hybridized to the microarrays at 42°C for 16 hours in a sealed hybridization cassette. To reduce the effects from individual differences, independent hybridizations were separately performed for three biological replicates, each of which was derived from 4 seedlings. Thus a total of 12 plants were used for each treatment. After hybridization and the ensuing washing steps, the chips were dried before scanning using the Agilent Scanner G2655AA (Agilent). Separate images were acquired, and the raw intensity values were obtained for Cy3 and Cy5. Missing spots and those covered by dust particles, with low signal intensity or in high-background areas were excluded from further analysis.

### Microarray Data Analysis

The resulting raw intensity data were uploaded to GeneSpring for background subtraction and normalization using the per-spot and per-chip intensity-dependent normalization (LOWESS) method. The normalized intensity values of duplicate spots were averaged, and the intensity ratio for each probe was calculated by ([averaged Cy3 intensity]/[averaged Cy5 intensity]). Significance analysis of microarrays (SAM) was applied across the control and treated samples in two-class test at a false discovery rate (FDR) of <0.05 [37]; 100 random permutations were used in this analysis. A transcript was considered as differentially expressed if it 1) passed the SAM test at FDR <0.05, and 2) had more than a 2-fold change. The differentially expressed transcripts were sequenced and deposited in GenBank. The BLASTX program was then used to search for *Arabidopsis* homologs at TAIR (http://www.arabidopsis.org) for these identified sequences. MeV v4.4 (http://www.tm4.org) was used for the hierarchical clustering analysis. The transcript profiling data for *Arabidopsis* were retrieved from the ME00328 dataset deposited at TAIR (http://www.arabidopsis.org).

### Real-time Quantitative PCR

Total RNA was extracted from the roots of 500 mM NaCl-treated *C. tagal* seedlings and the controls, adopting the same scheme as that used in the microarray hybridization. In each 25 µl real-time qPCR reaction, the cDNA template was initially generated from 7.5–10 ng total RNA. Each qPCR reaction also contained gene-specific primer pair (200 nM each), 1.5 µl EvaGreen (Biotium) dye, 0.5 µl ROX reference dye and 1 unit hot-start Taq DNA polymerase (TaKaRa). The primers used are listed in Table S1. The qPCR reactions were performed using the ABI PRISM1 7900 (Applied Biosystems) with the following program: hot start at 94°C and a 4-minute hold, followed by 40 cycles of 30 seconds at 94°C, 20 seconds at 55°C and 1 minute at 72°C. Both biological and technical replicates were included for a combination of genes and time periods. Non-template reactions in which sterile distilled water replaced the cDNA template were used as the controls. All of the data were analyzed using the SDS v2.2 software (ABI) based on the 2^-△△Ct^ method [38]. The resulting RQ value represented the relative expression level of a given gene by comparing with the unstressed control in the root tissue at a corresponding stress time point. The RQ values were log_2_-transformed for the ensuing analyses.

## Results

### Global Changes of Gene Transcription in *C. tagal* Under Salt Shock

Similar to other mangrove species, the seedlings of *C. tagal* are initially developed from viviparous propagules with comparatively low salinity [33]. When dropping from the mother trees and planting into ground that is immersed in seawater, the developing roots are shocked by the higher salinity of the seawater. To characterize the effects of salt shock on *C. tagal*, we performed a series of microarray experiments to monitor the transcript profiles using young roots that were shocked with 500 mM NaCl.

**Figure 1:**
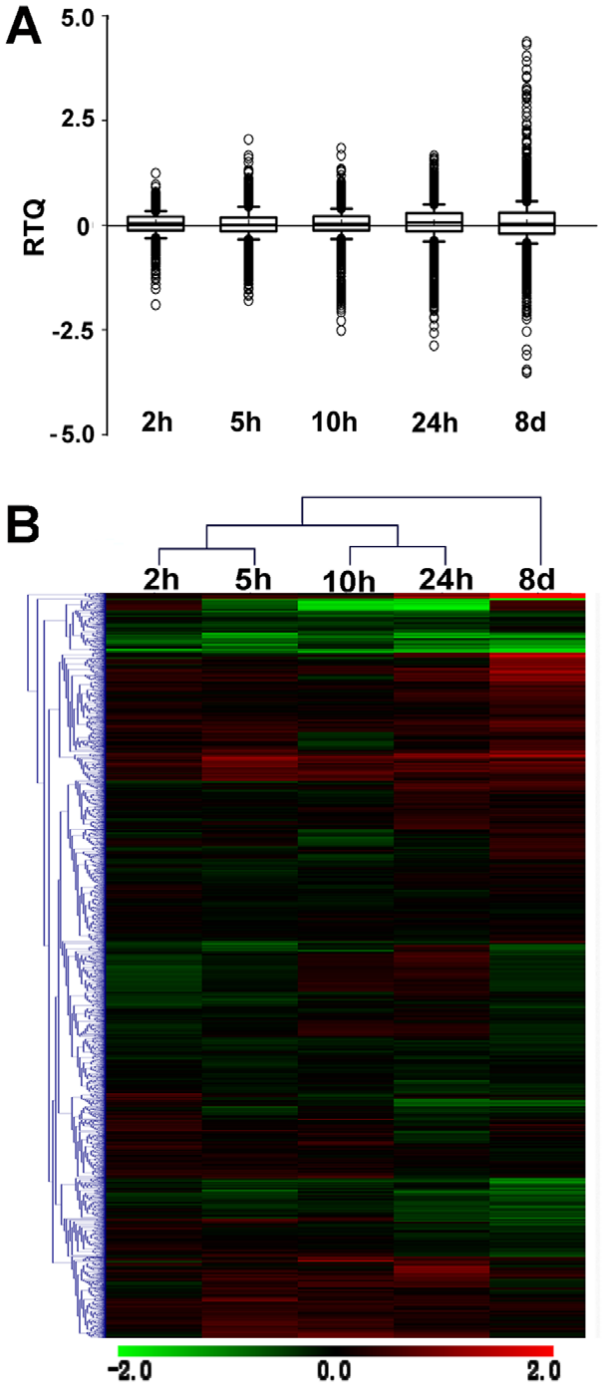
Global transcript profiles in the roots of salt-shocked *C. tagal* seedlings. A. The distribution spectrum of the RTQ (Relative Transcript Quantity) values of the whole set of investigated genes. The RTQ is defined as the log_2_-transformed fold change associated with each probe on the microarray chip. The RTQ values are ranked along the Y-axis, the box encloses those values between the 25^th^ and 75^th^ percentiles (the lower and upper quartiles, respectively), and the values between the 95^th^ and 5^th^ percentiles are enclosed by the up and down bars. The line near the middle of the box indicates the median value (the 50^th^ percentile). The circles indicate the outliers. B. Overview of the entire hierarchical clustering of the probes at all five time points. The heat maps display the transcript profiles by the log_2_-transformed fold changes on a color scale from green, indicating lower expression, to red, indicating higher expression, interpolated over black for the log_2_ (intensity ratio) = 0. Both the stress time points and probes/genes were clustered based on Euclidean distances.

We firstly surveyed the dispersion spectrum of the relative transcript quantity of each probe (RTQ, log2-transformed intensity ratio of each probe) at each time point using a box-plot chart (Figure 1, A). Assuming that the median level of RTQ under the unstressed condition was zero, it is clear that the value corresponding to the 2 h stress was slightly higher, whereas those corresponding to the last four stress treatments (5 h, 10 h, 24 h and 8 d) were close to zero. When considering the dispersion degree of the whole set of RTQ, however, most values at the 2 h stress time point fell into a very narrow range (Figure 1, A), indicating that the global transcription in the *C. tagal* roots was only slightly disturbed by the salt shock at this stage. When stress was prolonged, the RTQ values were spread out more, implying that transcription of most of the genes changed gradually over time, and consequently led to the expansion of the global transcription. Such a pattern, with an increasing trend of transcriptional activity, was inconsistent with the “burst” pattern in *Arabidopsis* (Figure S1) and other glycophytes [26].

A clustering analysis was also performed for the whole set of probes using the MeV v4.4 package. The output clearly indicated three clusters, 2 h and 5 h, 10 h and 24 h, and 8 d, representing three stress-duration-associated stages for the global transcript profiles (Figure 1, B).

### Identification and Functional Annotation of High-salinity-induced Differentially Expressed Genes (DEGs)

Differentially expressed genes (DEGs) were identified by performing the SAM statistics test at the significance level of FDR <0.05. From the whole set of the cDNA probes on the chip, we found 432 transcripts passing the test, but most of them displayed trivial changes in transcript abundance. Therefore, we also required a more than 2-fold change relative to the control at least at one time point in our survey. Consequently, 83 transcripts (Genbank Accession No. JK747783 - JK747799, JK747800 - JK747865) were identified to be significantly differential between treatment and control. These 83 sequences were then subjected to BLASTX searching against the *Arabidopsis* protein annotations at the TAIR website (http://www.arabidopsis.org), with a cutoff E-value of 10^−5^. Forty-three transcripts (52%) had significant matches in the *Arabidopsis* protein database, whereas 40 transcripts (48%) showed no hits. After the sequence assembly and subsequent manual adjustment based on the BLASTX results, we obtained 19 non-redundant annotated genes from the 43 transcripts that were homologous to *Arabidopsis* genes (Table 1; Table S2). The sequences with no matches in the *Arabidopsis* data set might be from the untranslated regions (UTRs) of a transcript or from specific genes that have no homologs in *Arabidopsis*. In summary, we obtained a total of 59 DEGs (19 annotated genes plus 40 no-hit sequences) that were responsive to salt shock in the salt-tolerant species, *C. tagal*. Among them, more genes (56) were down-regulated by the salt shock, which was consistent with the observations in *Arabidopsis* [39] to some extent in which the down-regulated transcripts in NaCl-treated roots were slightly more than those that were up-regulated.

**Table 1:** High salinity responsive genes in the roots of *Ceriops tagal*.

ID	*Arabidopsis* homolog	2 h	5 h	10 h	24 h	8 d
CtR_1	AT1G12110	1.04	1.92	**0.46**	**0.48**	**0.23**
	Nitrate transporter 1.1					
CtR_2	AT1G18210	0.8	**0.42**	0.75	**0.2**	**0.24**
	Putative calcium-binding protein CML26					
CtR_3	AT1G35140	0.58	**0.44**	**0.24**	**0.32**	**0.25**
	Phosphate-responsive 1 family protein					
CtR_4	AT2G18370	1.42	1.73	1.17	1.87	**2.43**
	Non-specific lipid-transfer protein 8					
CtR_5	AT2G21180	0.55	**0.41**	0.61	**0.42**	**0.42**
	Uncharacterized protein					
CtR_6	AT2G23810	0.95	0.72	0.95	0.66	**0.48**
	Tetraspanin 8					
CtR_7	AT2G24300	0.69	**0.35**	0.62	0.5	**0.35**
	Calmodulin-binding protein					
CtR_8	AT2G39380	**0.26**	0.54	**0.37**	**0.25**	**0.27**
	Exocyst complex component 7					
CtR_9	AT3G56360	0.86	0.88	0.81	0.56	**0.21**
	Uncharacterized protein					
CtR_10	AT4G07960	0.7	**0.41**	0.64	**0.46**	**0.42**
	Putative xyloglucan glycosyltransferase 12					
CtR_11	AT4G08950	0.54	**0.44**	**0.25**	**0.35**	**0.28**
	Phosphate-responsive 1-like protein					
CtR_12	AT4G25800	0.85	**0.42**	0.92	0.69	**0.44**
	Calmodulin-binding protein					
CtR_13	AT4G27960	**0.45**	**0.28**	**0.2**	**0.17**	**0.11**
	SUMO-conjugating enzyme UBC9					
CtR_14	AT4G33920	0.7	**0.44**	0.86	0.69	**0.5**
	Putative protein phosphatase 2C protein					
CtR_15	AT5G05960	0.9	1.03	1.2	1.24	**2.08**
	bifunctional inhibitor/lipid-transfer protein					
CtR_16	AT5G10695	**0.42**	**0.44**	0.66	**0.39**	**0.3**
	Uncharacterized protein					
CtR_17	AT5G57560	0.73	**0.31**	0.61	**0.38**	0.56
	Xyloglucan endotransglucosylase					
CtR_18	AT5G65730	0.68	0.75	**0.39**	**0.33**	**0.45**
	Xyloglucan:xyloglucosyl transferase					
CtR_19	AT4G25810	0.68	**0.32**	**0.17**	**0.13**	**0.09**
	Xyloglucan:xyloglucosyl transferase					

Note: Green indicates down-regulation while red indicates up-regulation.

**Figure 2:**
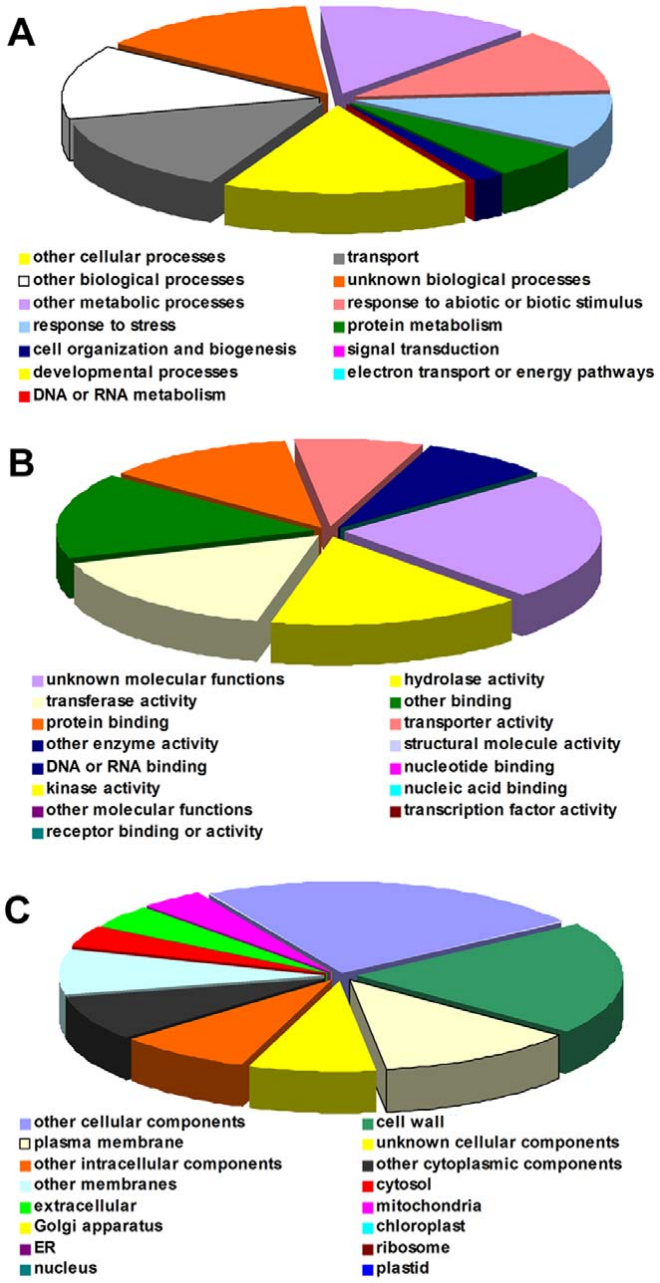
Functional classifications of *C. tagal* DEGs based on GO annotations. The DEGs were identified in the roots of *C. tagal* seedlings that were shocked with 500 mM NaCl, as described in the text. Shown are GOSlim-plant categories based on the ontology of (A) biological process, (B) molecular function and (C) cellular component.

GO annotations were assigned to each unique DEG according to the best hit in the BLASX search (Table S2) and then transferred into GOSlim-plant terms to generate a visible, intuitive distribution image of the genes. The distribution spectrum of the GOSlim plant categories, which was based on the ontology of biological process (BP), molecular function (MF) and cellular component (CC), are summarized in Figure 2.

### Transcript Profiling of DEGs

**Figure 3:**
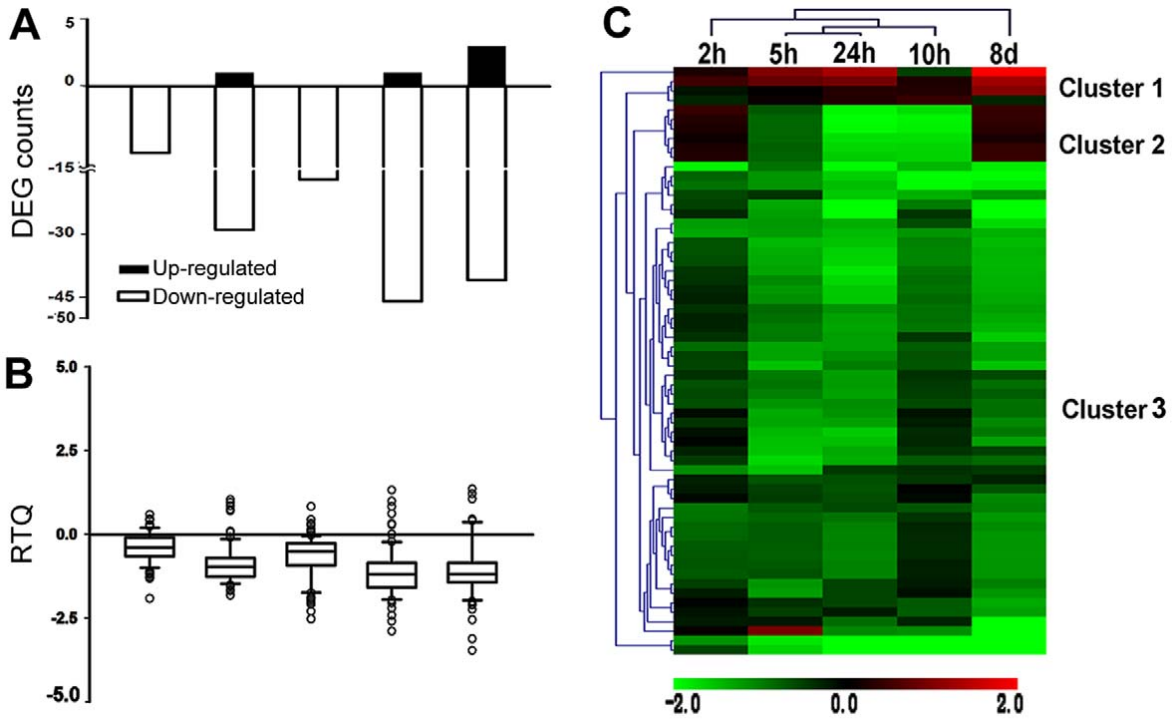
Transcript profiles of differentially expressed genes (DEGs). A. The dynamics of the number of differentially expressed genes (DEGs). Positive and negative values indicate the number of up- and down-regulated genes, respectively. B. Distribution spectrum of the RTQ (Relative Transcript Quantity) values of the DEGs. C. Transcript profiles of differentially expressed genes (DEGs). The heat maps illustrate the transcript profiles by the log_2_-transformed fold change on a color scale from green, indicating lower expression, to red, indicating higher expression, interpolated over black for the log_2_ (intensity ratio) = 0. Both the stress time points and probes/genes were clustered based on Euclidean distances.

The number of DEGs were 5, 30, 17, 47 and 44 for the 2, 5, 10, 24 hours and 8 days of stress, respectively, showing an increasing trend when the duration of the stress was extended (Figure 3, A). The median values of the expression level of the DEGs were notably lower on the box-plot chart, indicating a dramatic down-regulation in the salt-shocked roots of *C. tagal*. However, the dispersion of DEG expression had been increased gradually with time (Figure 3, B), which is consistent with the time-course dynamics of the dispersion of global expression (Figure 1, A).

Hierarchical clustering for the DEGs generated 3 clusters (Figure 3, C). Clusters 1 and 3 contained genes that were up- or down-regulated over all of the time points, respectively, whereas Cluster 2 contained those genes that were down-regulated at two or three time points. Two genes involved in lipid transport were included in Cluster 1 and were dramatically activated after 8 days of salt stress. Four genes annotated as responsive to abiotic stimuli were suppressed by salt shock and were grouped into Cluster 3, two of which were related to responses to water deprivation.

### Real-time Quantitative PCR for Selected DEGs

**Figure 4:**
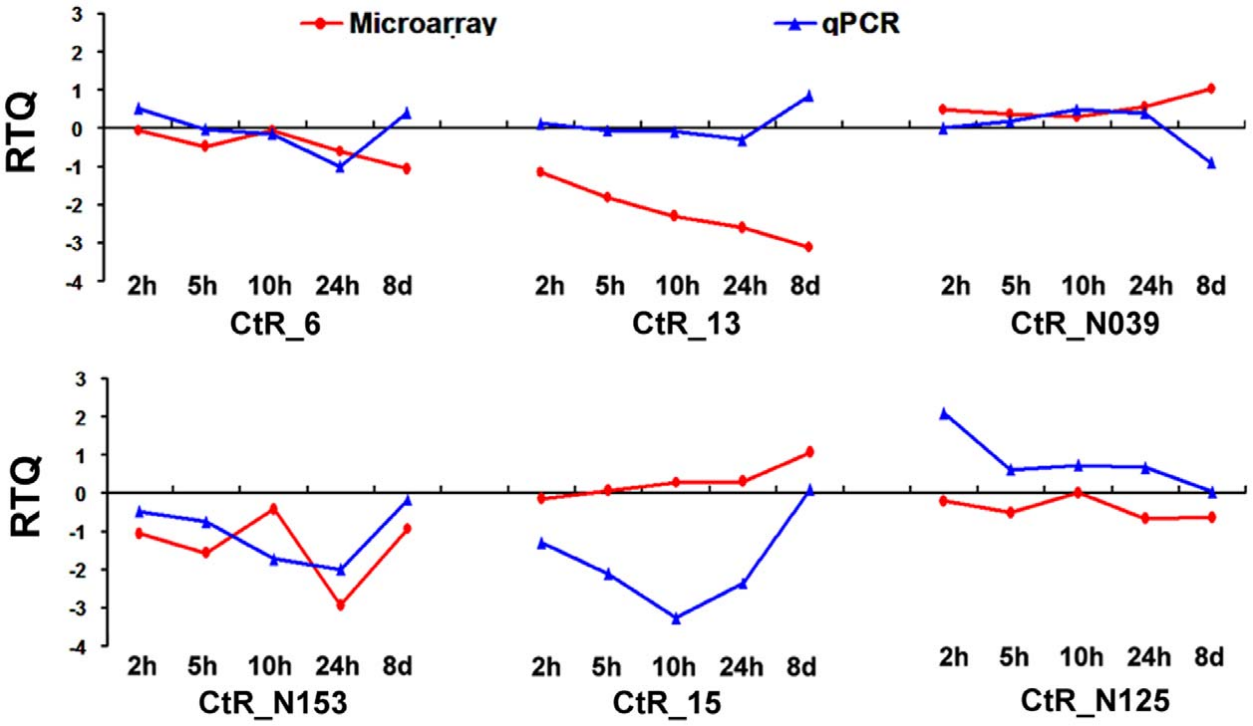
Real-time quantitative PCR assay for six randomly selected genes. Total RNA was extracted from the roots that were stressed with 500 mM NaCl for 2, 5, 10, and 24 hours and 8 days (192 hours). The collection of control samples and the RNA extractions were performed in parallel. The RTQ (Relative Transcript Quantity) of each gene at each time point is shown on the Y-axis by the log_2_-transformed values of the fold changes in the microarray and the RQ values from real-time qRT-PCR experiments, respectively. CtR_6, CtR_13 and CtR_15 were selected from DEGs, while CtR_N039, CtR_N125 and CtR_N153 from non-DEG set.

To validate the microarray results, real-time quantitative analyses were performed on six randomly selected transcripts, three from the DEG set and three from non-DEG set (Table S1). Our results indicated that the transcriptional changes demonstrated by qPCR were in accordance with those from microarray assays at approximately 57% of the time points (Figure 4). Disagreements were also observed, which probably were caused by the discrepancy in the detection sensitivity between the two platforms [40]. Considering inevitable technical variation associated with the cDNA microarray techniques [41]–[43], more replicates may be required for reliable detection of the DEGs, especially for those are low or high in transcriptional abundance. In future, new techniques such as next-generation sequencing would be a promising tool for unbiased detection of DEGs [44].

### Pair-wise Comparison between Homologous Genes in Ceriops tagal and the Model Plant, Arabidopsis

**Figure 5:**
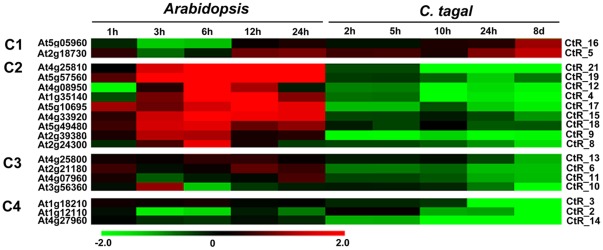
Pair–wise comparison between predicted homologs from *C. tagal* and *Arabidopsis*. Microarray data of *Arabidopsis* genes were retrieved from the ME00328 microarray dataset (available at the TAIR website). The *K*-means clustering of predicted homologous gene pairs generated four groups that indicate gene pairs with divergent (C1, C2, and C3) or conserved (C4) regulation patterns between *C. tagal* and *Arabidopsis*, respectively. Red, up-regulation; green, down-regulation; black, no change. *Arabidopsis*, *Arabidopsis thaliana*; *C. tagal*, *Ceriops tagal*.

From the microarray dataset available from TAIR, we retrieved the data of 18 *Arabidopsis* homologs of *C. tagal* DEGs. Using *K*-means clustering analysis which followed by manually adjustment, four separated groups were generated, as shown in Figure 5. Groups of C1, C2 and C3 contained more than 80% of the *C. tagal* DEGs (15 genes) and highlighted the differences in the salt-induced responses between *C. tagal* and *Arabidopsis*. The expression of paired genes in Group C3 varied very slightly between *C. tagal* and *Arabidopsis* within the first 24 hours; however, the *C. tagal* homologs were dramatically repressed after 8 days. Group C4 consisted of three pairs of genes that were annotated as “lipid transport” or “response to water deprivation”, and these genes were regulated in a conservative manner in the two species (Figure 4). We further retrieved the expression data of the *Arabidopsis* homologous genes using Genevestigator 3.0 [45] and performed a comparison with the *C. tagal* DEGs manually. It indicated that the proportion of oppositely regulated gene pairs approached 45%. Taking together, these observations indicated that the salt shock induced differential responses in the roots of *C. tagal* and *Arabidopsis* and *C. tagal* would cope with stress of high salinity in a manner that is different from *Arabidopsis*.

## Discussion

### Stabilization-prone Transcriptional Homeostasis in Halophytic *C. tagal*

The adaptation of mangroves to saline environments is related to transcriptional regulation. In a recent study on the transcriptomes of two mangrove species, *Rhizophora mangle* and *Heritiera littoralis*, the authors observed that the distributions of the GO lineages and KEGG pathways of these two mangroves were similar to each other but differed substantially from those of model plants [46], suggesting a unique mangrove lifestyle. *Laguncularia racemosa*, also a mangrove species, shows little genetic but large epigenetic differences between populations occurring in naturally contrasting habitats, at a riverside or near a salt marsh, implying that epigenetic variations in natural plant populations have an important role in helping the individuals to cope with different environments [47].

*C. tagal* is a salt-tolerant species and has many typical features that are associated with the adaptation to saline environments. As stated in the Introduction section, rapid and successful rooting into saline soils is one of the key steps for survival under such challenging environments. In the present study, we found that the global transcription in the roots of *C. tagal* changed only slightly within the first 24 hours of salt stress (Figure 1, A), presenting a comparatively stable status. However, the degree and extent of the alterations in gene expression gradually increased when the salt shock was extended to eight days (Figure 1, A), suggesting a systematic response to the long-term stimulus. This conclusion, independent of the analysis methods, holds whether the time-course dynamics of the number of DGEs or that of the expression dispersion of the DEGs were evaluated (Figure 3, A, B).

An explanation for this pattern is that the expression in the roots of *C. tagal* was constrained at the initiation of the salt stress, with the purpose of avoiding that 1) the plants become over-sensitive to the environmental challenges, which might perturb the global transcription, and 2) too many genes, and therefore the biological processes, were affected by the sublethal salinity at an early stage. Through such a mild regulation that combined a subtle adjustment with the systematic activation of transcription, *C. tagal* could cope with the stress of high salinity, possibly by a time lag between the salt shock and response. This regulation, termed stabilization-prone transcriptional homeostasis in this study, could confer a greater capacity on the plants to adapt to the environments.

Such a pattern of transcriptional homeostasis under salt stress seems to be linked to salt-tolerant lifestyles in ice plant [26] and salt-acclimated yeast [48] in addition to *C. tagal*. In contrast, salt-sensitive species, such as *Arabidopsis* (Figure S1) and rice [26], show a transcriptional “burst” after 6 hours of salt stress, implying a transient irritability in the response to salt stress. The observed differential regulation on the global transcription between glycophytes and halophytes suggests that salt-induced responses in halophytes may be tightly associated with the adaptation to the saline environment. However, it should be noted that the differential regulation can also attributed to genetic divergence and/or phenotypic plasticity; thus, more data are needed to test this hypothesis.

We reanalyzed the microarray data reported by Kreps et al. [19] and found that the GO term “response to stress” was significantly overrepresented at both 3 h and 27 h after the perception of salt stress in *Arabidopsis*, which could help resist the challenge of high salinity. We performed the same analysis using the *C. tagal* DEGs as queries, aiming at identifying the major gene groups that potentially function in transcriptional homeostasis. However, the GO term “response to stress” was not significantly over-represented as expected (data not shown), probably due to the small number of DGEs. An alternative explanation for the un-representation of stress-responsive gene groups is that they would not be disturbed by salt shock in the roots of *C. tagal*. If true, such differences in the gene enrichment pattern could reinforce the view that high salinity would not massively perturb gene transcription in the roots of *C. tagal* and that the transcriptional regulation under salt shock in mangrove species could not be attributable to those generally acknowledged stress-related pathways. More evidences were also found on the transcriptional divergence between the homologous genes of *C. tagal* and *Arabidopsis* (Figure 5), which further suggested mechanistically distinct regulation between these two species.

### 4.2 Transcriptional Divergence between *C. tagal* and Arabidopsis

Interspecies divergence in transcription factor binding sites and *cis*-regulatory elements have been observed previously and are suggested to contribute to regulatory evolution [49]–[51]. From the 18 homologous gene pairs identified in this study, we found that approximately 80% of the *C. tagal* genes were regulated by high salinity in a way that was opposite to that in *Arabidopsis* (Figure 5). One cause for such substantial divergence in transcriptional phenotypes is likely that the high salinity would act as a selective pressure in the environment. However, we cannot rule out the possibility that the divergence would be caused by genetic divergence. Large differences can be expected between distant species, such as *C. tagal* and *Arabidopsis*, as phenotypic difference in gene expression is a function of phylogenetic divergence under the neutral prediction [11]. Thus, transcriptional divergence between homologs across species could not reflect the real differences that result from salinity-driven selection if the effect of the phylogenic distance is not eliminated. Furthermore, because divergence between species differs from gene to gene due to evolutionary constraints [4], investigation on the divergence of gene structure, function, and transcription at the individual gene and the whole-genome levels is required to dissect the mechanism of transcriptional divergence between homologs in distantly related species.

Across-taxa comparisons also permit the identification of conserved transcriptional profiles and could uncover molecular mechanisms that are responsible for fundamental biological processes [52]. An interesting result from our analysis is that some genes annotated as lipid transport or responding to water deprivation are highly conserved in the expression patterns between *C. tagal* and *Arabidopsis* (Figure 5). Either stabilizing selection or evolutionary constraints are possible causes of such phenotypic conservation [53], [11]. However, it is difficult to attribute the observed conservation in the present study to stabilizing selection because the interaction between stabilizing selection and drift may increase divergence or constrain variation, and, moreover, such interactions become more complex as the phylogenetic distance increases [53], [11]. More powerful methods are needed to determine whether these conserved genes have experienced selection.

In summary, microarray technology is a stand-by and powerful approach in ecogenomic studies [54]. By using such a technique, comparative analyses in terms of ecology and evolution at the genomic scale can be performed. In this study, we applied microarray technology to investigate the transcriptional profiles of *C. tagal*, a mangrove species inhabiting intertidal zones, and conducted a comparative analysis with the model glycophyte, *Arabidopsis*. The results suggest that transcriptional homeostasis might be a specific salt-related response in *C. tagal* and might be associated with the adaptation to high-salinity environments. Our comparison between homologs in *C. tagal* and *Arabidopsis* allows the identification of candidate genes under selection, which may provide a basis for future studies.

## Supporting Information

Figure S1 **Salt stress induced transcription dispersion in roots of** ***Arabidopsis*.**
(PDF)

Table S1 **Primer pairs used in real-time quantitative PCR assay.**
(PDF)

Table S2 **A complete sheet listing the 59 DEGs and GO annotations.**
(XLS)
