# Supplementary material for: Transcriptional Homeostasis of a Mangrove Species, Ceriops tagal, in Saline Environments, as Revealed by Microarray Analysis
Source: PLoS One. 2012 May 4;7(5):e36499. doi: 10.1371/journal.pone.0036499 (PMC3344879; doi:10.1371/journal.pone.0036499)

Figure S1 **Time-course profile of distribution of relative expression level in root of salt stressed *Arabidopsis*.**

Microarray dataset (ME00328) were retrieved from publically access resources at TAIR (http://www.arabidopsis.org/info/expression/ATGenExpress.jsp). Experimental description can be found both in Kilian, et al. 2007 (*Plant J* (2007) 50, 347–363, doi: 0.1111/j.1365-313X.2007.03052.x) and at TAIR website. Normalized data were used in the present study to plot the distribution of log2-transferred changed fold (Y-axis, Relative expression level) against the stress time periods (X-axis). It shows that an obvious "burst" presented at the time point of 6h, indicating a transient expansion of the global transcription level.

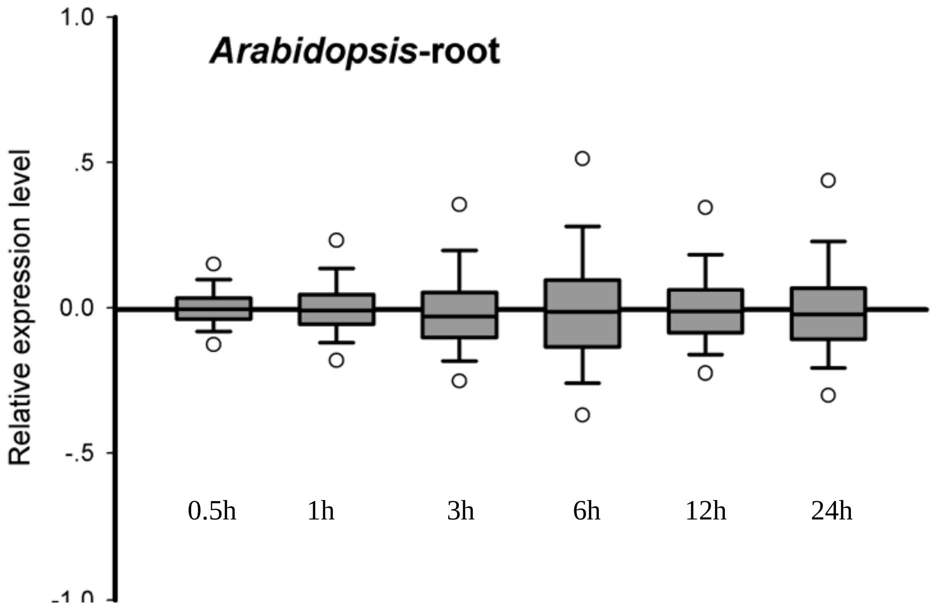

Supplement: Figure S1 — Salt stress induced transcription dispersion in roots of Arabidopsis. (PDF) [file pone.0036499.s001.pdf]
