# Supplementary material for: Transcriptional Homeostasis of a Mangrove Species, Ceriops tagal, in Saline Environments, as Revealed by Microarray Analysis
Source: PLoS One. 2012 May 4;7(5):e36499. doi: 10.1371/journal.pone.0036499 (PMC3344879; doi:10.1371/journal.pone.0036499)
Supplement: Table S1 — Primer pairs used in real-time quantitative PCR assay. (PDF) [file pone.0036499.s002.pdf]

Table S1 Primer pairs used in quantitative PCR

Gene ID	Forward Primer	Reverse sequence
18S rRNA	GAACGGCCATGCACCACCAC	GGAAGGGCACCACCAGGAGT
CtR_N125	TCTCGTTTTCCTCTTCACTT	AGGTCGTCCCTGCTTATC
CtR_6	TGTTACCCAGTTCTACAACGAGCA	TTTTCCCAGGCATCACAGTCAG
CtR_N039	CCTTTTCCTAGGAGTTACTATCGT	AAAGGCAGCGGCAGAGAA
CtR_N153	ACATTTACTTGCCTCGGGATT	AACCGTTACAGGAAACTTGAGGA
CtR_15	AGCCTGCCTCTGTGCTGTTA	GCTGCATACTCAATGTCTTCCC
CtR_13	TTTAAGCCTCCCAAGGTAGCC	AAAGGGTCATCAGGGTTCG
